# Space biology as a catalyst for the next biotech and longevity revolution

**DOI:** 10.1113/EP093956

**Published:** 2026-08-18

**Authors:** Carina Kern, Matthew Cook, Elizabeth Reynolds, Anuj Ashar, Keith Siew, Ian R. D. Johnson, Nikodem Grzesiak, Louise Jopling, Meganne Christian, Rafal Libera, Immanuel Gartner, Miguel J. S. Ferreira, Aliasger K. Salem, William Birch, Nasreen Dhanji, David C. Cullen, Jorge M. Sampaio, Diogo Oliveira, Stuart Catchpole, Rosie Richardson, Dominykas Milašius, Damian Miles Bailey

**Affiliations:** ^1^ LinkGevity Babraham Research Campus Cambridge UK; ^2^ UK Space Agency UK; ^3^ Space Age, Translational Research Institute for Space Health (TRISH) USA; ^4^ Asteria Space UK; ^5^ London Tubular Centre, Royal Free Hospital London UK; ^6^ UCL Centre for Kidney and Bladder Health University College London London UK; ^7^ Telespazio Belgium S.R.L. for the European Space Agency Noordwijk Netherlands; ^8^ School of Pharmacy and Biomedical Sciences, College of Health Adelaide University Adelaide Australia; ^9^ Babraham Research Campus Cambridge UK; ^10^ Office of the Chief Medical Officer BK Klosterneuburg Dukes Klosterneuburg Austria; ^11^ Gartner.FIT Vienna Austria; ^12^ Medical Directorate FIBA 3X3 Vienna Austria; ^13^ Space Applications Services NV/SA Sint‐Stevens‐Woluwe Belgium; ^14^ University of Iowa Iowa City Iowa USA; ^15^ Satellite Applications Catapult, Oxfordshire UK; ^16^ Cranfield University, Cranfield UK; ^17^ Loka Los Altos California USA; ^18^ Space East, Suffolk County Council, East of England UK; ^19^ School of Life Sciences Anglia Ruskin University Cambridge; ^20^ Delta Biosciences Lithuania; ^21^ Neurovascular Research Laboratory, Faculty of Life Sciences and Education University of South Wales Pontypridd UK; ^22^ Bexorg Inc New Haven Connecticut USA

## 1 SPACE BIOLOGY AT A TRANSLATIONAL TURNING POINT

A recent conference convened by the UK Space Agency, LinkGevity, Space East, Loka, Amazon Web Services and Delta Biosciences brought together researchers, clinicians, astronauts, athletes, technologists, investors and policymakers to examine how space biology is emerging as a driver of innovation across the life sciences. The conference was titled ‘The Next Frontier: Biotech Beyond Earth Conference’ (agenda: Table 1 and https://luma.com/fsh1jy9r). Held at the Babraham Research Campus, situated within the heart of the Cambridge life science cluster, the meeting was well placed within one of the UK's leading bioscience campuses, where academic discovery and industrial innovation coexist in unusual proximity.

**TABLE 1:** The next frontier: Biotech beyond earth conference overview.

**About the Event**
As humanity prepares for long‐duration space missions, one of the greatest obstacles lies not only in rockets and satellites but in our own biology. Space travel exposes the human body to unique challenges, including microgravity, hypo‐gravity and cosmic radiation, which accelerate aging and cause organ damage. 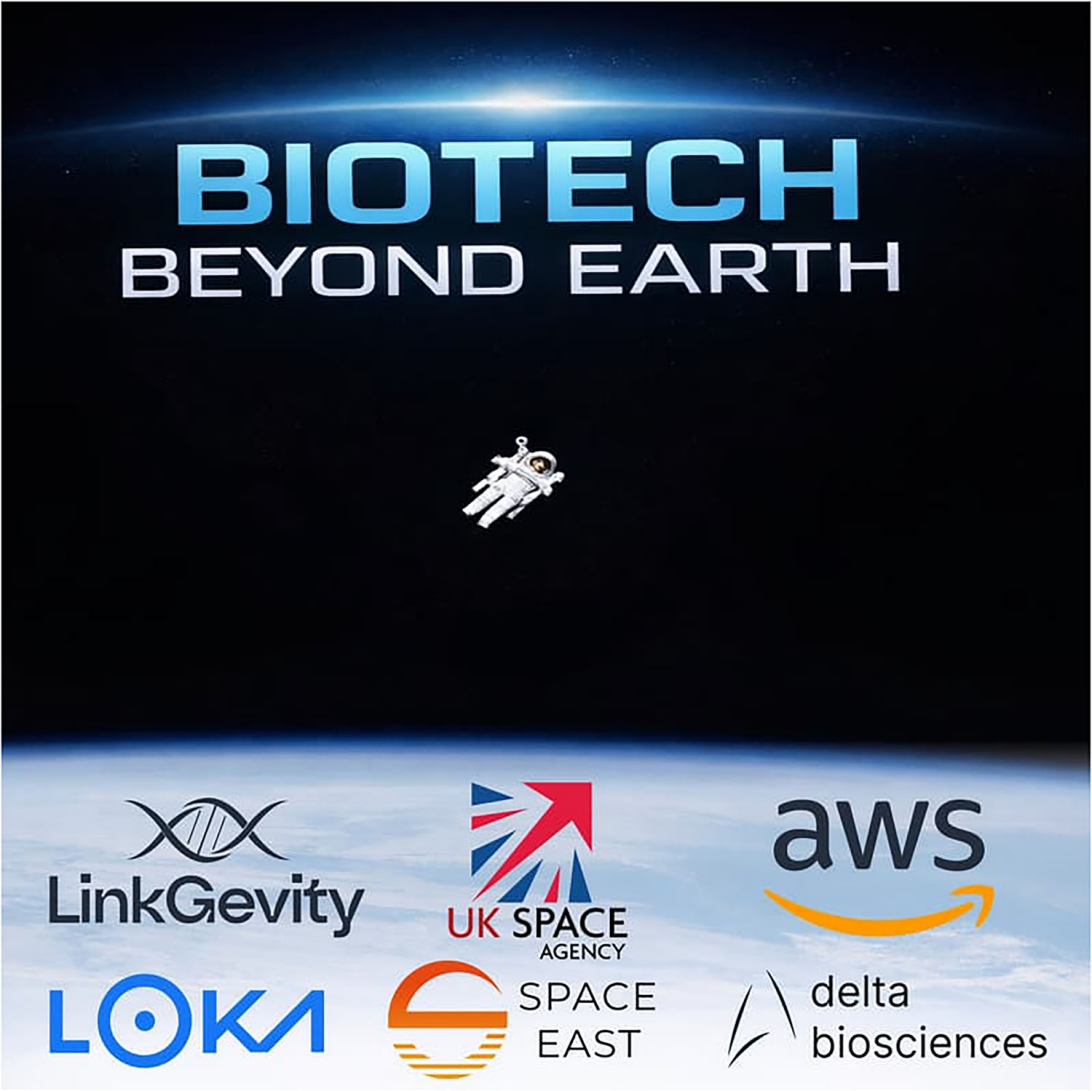 This conference will bring together leading experts to explore cutting‐edge research and innovations that could make extended space travel a reality while shedding light on the invaluable lessons space biology offers for life on Earth. From addressing age‐related conditions to tackling kidney disease to preserving brain function, the physiological insights gained from space are driving breakthroughs in healthcare here at home. This is a conference aimed at showcasing how space biology is driving biotech breakthroughs and how life sciences can be leveraged to drive the Space Health agenda. It will be held at the Babraham Research Campus, a major life sciences research and innovation campus in Cambridge, UK. The event is being organised by LinkGevity, a Cambridge‐based, AI‐enabled drug discovery company focused on aging and age‐related deterioration, and Delta Biosciences, a developer of ultra‐miniaturised screening technologies for drug discovery and space health applications. The strategic partners of the conference are the UK Space Agency and Space East.
**Agenda**
1:30–1:45 pm – Arrival and Registration with Refreshments1:45–2:00 pm – Welcome Remarks Matthew Cook, Head of Space Exploration, UK Space Agency Dr Louise Jopling, Chief Scientific and Innovation Officer, Babraham Research Campus Stuart Catchpole, Regional Director, Space East
2:00–2:30 pm – Testing the Limits of Human Endurance – How Best to Manage Extreme Environments? Prof. Damian Bailey, Chair, Life Sciences Working Group, European Space Agency Dr Meganne Christian, ESA Astronaut; Exploration Commercialisation Lead, UK Space Agency Dr Immanuel Gartner, former pro athlete, Medical Director, Prevention Specialist, M.D.
2:30–3:15 pm – Panel I: Overcoming Biological Hurdles and Pushing New Frontiers in Space Dr Keith Siew, NASA SciX Lead Ambassador & Senior Research Fellow, UCL Prof. David Cullen, Professor of Astrobiology and Space Biotechnology, Cranfield University Tuva Atasever, Astronaut, Turkish Space Agency Dr Ian Johnson, Senior Coordinator for Life Sciences, European Space Agency Dr Aliasger Salem, University of Iowa – Professor / Associate Vice President for Research
3:15–3:30 pm – Refreshments Break3:30–3:45 pm – LinkGevity – A First in Class Anti‐Necrotic to Revolutionise the Treatment of Ageing on Earth and Beyond Dr Carina Kern, CEO and Founder, LinkGevity
3:45–4:30 pm – Panel II: How Advances in Space Can Reshape Innovation on Earth Elizabeth Reynolds, Executive Director, Space Age, Translational Research Institute for Space Health (TRISH) Dr Carina Kern, CEO and Founder, LinkGevity Prof. Damian Bailey, Chair, Life Sciences Working Group, European Space Agency Dr Christopher Puhl, Business Development Manager, Exobiosphere
4:30–5:15 pm – Panel III: Investment Opportunities in Space for Pharma & Biotech Anuj Ashar, Founder & General Partner, Asteria Space William Birch, Strategic Account Director: Space, Satellite Applications Catapult Dr Miguel Ferreira, Biosciences Space Business Developer, Space Applications Services NV/SA Diogo Caldas de Oliveira, Machine Learning Team Lead, Loka Inc. Dominykas Milasius, Co‐founder, Delta Biosciences
5:15 pm – Cream Tea (scones with clotted cream and jam)6:00 pm – Close
**Location**
Babraham Research Campus Babraham, Cambridge CB22 3AT, UK

**FIGURE 1:**
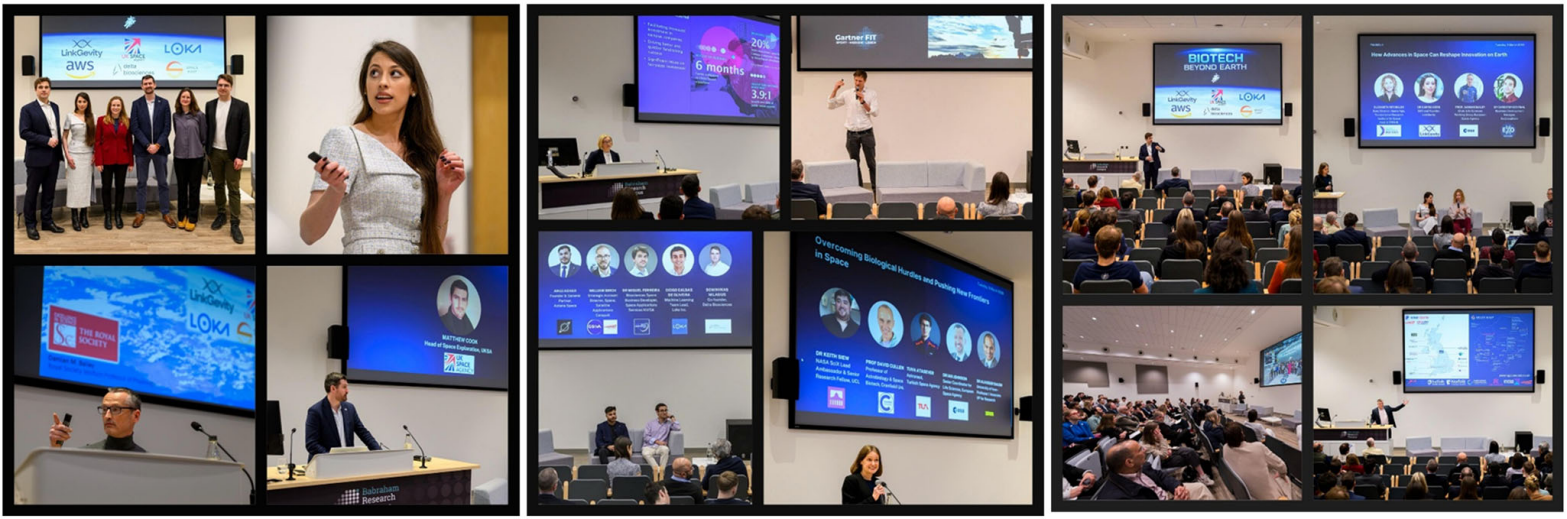
Panel discussions at ‘The Next Frontier: Biotech Beyond Earth’ conference bringing together diverse voices across science, medicine, technology and investment, underscoring the interdisciplinary momentum now driving space biology.

The message was clear: space is no longer a remote scientific frontier divorced from everyday medicine, but a powerful biological laboratory and translational opportunity. As the field matures, space biology is beginning to connect extreme physiology, biotechnology, healthy ageing and precision medicine within a common scientific and commercial framework (Bailey, 2026; Garrett‐Bakelman et al., 2019; Kern & Siew, 2026; Mason et al., 2024; Rutter et al., 2024). Figure 1 captures one of the focused panel discussions (see later), reflecting the strongly interdisciplinary and translational character of the meeting.

As ambitions expand towards longer missions to the Moon, Mars and beyond, the primary limiting factor is increasingly recognised to be human biology rather than solely engineering. Human spaceflight exposes astronauts to the full force of the space exposome: cosmic radiation, isolation and confinement, distance from Earth, hostile closed environments, and altered gravity fields. These hazards accelerate biological decline, impair organ function and push the limits of human adaptation (Bailey, 2026; Bailey et al., 2025; Goswami et al., 2026). Yet these same stressors also create an extraordinary experimental model. By compressing complex physiological strain into an experimentally measurable and repeatable timeframe, space offers a powerful means of interrogating mechanisms of dysfunction, resilience and repair that are directly relevant to disease and ageing on Earth and translating these insights into targeted therapeutic strategies.

The conference captured this translational breadth explicitly. Sessions ranged from human endurance in extreme environments, the biological hurdles of long‐duration spaceflight, space‐enabled innovation on Earth – including Earth‐based advances that may enable longer‐duration missions – and the investment case for biotech and pharma. What emerged was not simply a showcase of new science, but the articulation of a coherent and interconnected ecosystem in which physiology, engineering, omics, diagnostics and venture strategy are beginning to align (Bailey & van Ombergen, 2026; Camera et al., 2024; Garrett‐Bakelman et al., 2019; Scott et al., 2022). The UK is one of the world's strongest biomedical landscapes, and well‐positioned to contribute to this convergence.

## 2 SPACE AS A STRESS TEST FOR HUMAN BIOLOGY

One of the most important ideas to emerge from the meeting was that spaceflight should not be viewed solely as a collection of hazards to be mitigated, but as a biologically revealing stress model that directly informs the design of effective countermeasures and the development of therapeutics. Space does not create entirely new biology; rather, it exaggerates familiar physiological stress pathways across multiple systems at once (Bailey et al., 2025; Camera et al., 2024; Garrett‐Bakelman et al., 2019; Rutter et al., 2024; Scott et al., 2022).

Human spaceflight disrupts nearly every major physiological system. Reduced gravitational loading drives rapid musculoskeletal deterioration, cardiovascular deconditioning and fluid redistribution. Ionising radiation induces DNA damage and mitochondrial dysfunction, while circadian disruption and isolation affect cognition and mental health (Bailey et al., 2025). Many of these responses resemble, and often accelerate, processes associated with ageing and chronic disease on Earth.

Space effectively compresses years of decline into months, allowing researchers to observe early drivers of tissue degeneration, inflammation, immune perturbation and organ dysfunction. These insights are highly relevant to osteoporosis, kidney disease, immune dysfunction, cardiovascular decline, neurodegeneration and frailty. Particularly compelling was the framing of space as a ‘fast‐forward’ model of ageing and disease, in which vascular dysfunction, musculoskeletal unloading, systemic oxidative–inflammatory–nitrosative stress, cellular necrosis and degeneration, mitochondrial perturbation, neurovestibular disturbance, circadian disruption, and psychosocial strain converge in accelerated form (Bailey et al., 2020; Camera et al., 2024; Garrett‐Bakelman et al., 2019; Kern, Bonventre et al., 2025; Scott et al., 2022).

In this respect, space biology is not peripheral to medicine. It offers a systems‐level view of human decline that is difficult to reproduce in conventional laboratory or clinical settings. This logic resonates strongly with integrative human physiology more broadly, where the aim is to understand how stressors such as hypoxia, exercise, radiation, confinement, altered gravity and environmental extremes challenge homeostasis at the level of the whole organism. Time and again, the most informative physiology emerges under stress, not rest. Extreme environments expose regulatory limits, reveal latent vulnerabilities and illuminate the compensatory mechanisms that preserve function (Bailey, 2026; Bailey & van Ombergen, 2026; Vrdoljak et al., 2026). Taken together, it was made clear that extreme physiology provides a vital conceptual bridge between space exploration and terrestrial medicine.

## 3 EXTREME PHYSIOLOGY, SPACEFLIGHT AND THE BIOLOGY OF AGEING

A recurring motif across sessions was the overlap between space‑induced stress and biological ageing. Spaceflight‐associated stressors, including hypoxia, radiation exposure, isolation, circadian disruption and physical deconditioning, resemble or amplify core features of biological ageing. Systemic oxidative–inflammatory–nitrosative stress, endothelial dysfunction, impaired mitochondrial bioenergetics, sarcopenia, insulin resistance and neurocognitive decline are not merely operational concerns for astronauts; they are central features of age‐related decline on Earth. By provoking these phenotypes rapidly and in combination, space enables researchers to interrogate mechanisms that would otherwise unfold slowly and heterogeneously across decades (Bailey, 2026; Camera et al., 2024; Kern, Bonventre et al., 2025; Scott et al., 2022).

This theme was brought into sharp focus during the opening session, ‘Testing the limits of human endurance—how best to manage extreme environments?’, which placed the human body at the centre of exploration. The discussion moved beyond survival and towards a more important physiological question: how do we preserve function, adaptability and resilience when multiple regulatory systems are pushed simultaneously towards their limits? That question is no less relevant to older adults, patients with chronic disease and overstretched healthcare systems on Earth as it is to crews travelling to the Moon or Mars (Bailey, 2026; Bailey & van Ombergen, 2026).

## 4 LONGEVITY SCIENCE BEYOND EARTH

Longevity was a major thread running through the meeting. The UK is already recognised for strengths in ageing biology, regenerative medicine and data science, and space‐based research platforms add a powerful translational dimension to that portfolio. Under the extreme conditions of spaceflight, researchers can test hypotheses about ageing mechanisms more rapidly, identify vulnerable pathways earlier and de‐risk therapeutic development more efficiently. For longevity science, space is not an abstraction; it is an amplifier (Bailey, 2026; Mason et al., 2024; Rutter et al., 2024).

Crucially, several contributions emphasised that longevity should not be framed narrowly as lifespan extension, but as preservation of integrated physiological capacity across the life course. That distinction matters. The central challenge facing ageing societies is not simply how long people live, but how well. Space biology intersects with longevity science most powerfully when it enables targeted interventions that sustain vascular integrity, musculoskeletal competence, cognitive performance, metabolic flexibility and organ resilience (Camera et al., 2024; Garrett‐Bakelman et al., 2019; Rutter et al., 2024; Scott et al., 2022).

The convergence becomes especially compelling when considering the same physiological systems destabilised in orbit are often those that fail first with advancing age on Earth. Space therefore provides an unusually stringent proving ground for countermeasures, one in which candidate drugs, diagnostics, biomarker platforms and monitoring systems can be challenged against accelerated multisystem stress. In that sense, space is not merely relevant to longevity science; it is strategically catalytic (Bailey et al., 2025; Camera et al., 2024; Mason et al., 2024; Scott et al., 2022).

## 5 FROM SPACE PHYSIOLOGY TO THERAPEUTIC INNOVATION

The conference highlighted how AI‐enabled drug discovery, omics technologies and ultra‐miniaturised screening platforms are making sophisticated biological experimentation in space increasingly feasible. Microgravity and radiation can unmask cellular processes that remain obscured under Earth‐normal conditions, thereby exposing mechanisms and targets that may be therapeutically actionable. Emerging efforts to develop compact, autonomous and high‐content experimental systems for use in flight reinforced the sense that space biology is becoming technically mature as well as conceptually important.

This translational potential was illustrated by a dedicated spotlight presentation by Dr Carina Kern who introduced the UK Space Agency supported AstroSANITAS (Stable Anti‐Necrotic for In‐space Tissue Augmentation and Survival) programme, centred on a first‐in‐class anti‐necrotic therapeutic targeting uncontrolled cell death (necrosis) – the central mechanistic basis of degeneration across biological systems and conditions, including kidney disease, neurodegeneration, cardiovascular pathology and ageing (Kern, Bonventre et al., 2025). Early data indicating substantial protection against tissue degeneration were presented alongside plans for in‐orbit validation, positioning space as a uniquely powerful environment to test interventions that aim to preserve cellular and organ integrity under extreme stress. Crucially, such terrestrially developed therapeutics will play a key enabling role in space exploration itself, helping to mitigate cumulative tissue damage, extend physiological resilience, and support the feasibility of longer‐duration missions beyond low Earth orbit.

Overall, this convergence is reshaping drug discovery. Insights generated in space are informing the development of interventions designed to prevent tissue injury, preserve organ function and enhance cellular resilience. More importantly, these approaches dissolve traditional boundaries between ‘space medicine’ and terrestrial healthcare, positioning space biology as a driver of mainstream biomedical innovation. The emphasis was on integrating the discovery of new molecules with the identification of actionable targets and mechanisms of degeneration and resilience under integrated whole‐organism stress, a core phenotypic principle of the integrome (Bailey, 2026; Kern, Jenkins et al., 2025).

Alongside development of novel therapeutics, technologies developed for autonomous experimentation, remote monitoring and constrained extremes are directly relevant to personalised medicine, remote care and healthcare delivery in resource‐limited settings on Earth. This was explored particularly well during the panel discussion, ‘How advances in space can reshape innovation on Earth’, which focused on practical translation rather than abstract promise. The deliberately provocative question, ‘what's space ever done for us?’, forced the panel to articulate concrete terrestrial dividends from space research (Bellisle et al., 2020; Roda et al., 2018; Scott et al., 2022).

Two themes stood out. First, extreme environments such as spaceflight, hypoxia, radiation and isolation were framed as powerful ‘fast‐forward’ models of human ageing and disease, capable of accelerating innovation in vascular, neurodegenerative, renal and cardiometabolic medicine. Second, the panel explored translational bioengineering from orbit to clinic. Technologies developed to monitor astronauts, including omics platforms, wearable biosensors, autonomous diagnostics and intelligent decision‐support systems, have obvious relevance to precision medicine, home‐based monitoring, remote care and healthcare resilience in underserved or operationally constrained environments (Bailey, 2026; Bellisle et al., 2020; Camera et al., 2024; Roda et al., 2018; Rutter et al., 2024; Vinken et al., 2025).

What made these discussions especially valuable was that they moved beyond simplistic narratives of ‘spin‐out’ and instead highlighted conceptual translation: new ways of measuring integrative physiology, predicting deterioration, personalising intervention and managing health when staffing, infrastructure or specialist oversight are limited. In a world of ageing populations, multimorbidity and rising healthcare strain, that logic is increasingly difficult to ignore (Ciofani et al., 2025; Scott et al., 2022).

## 6 PANELS, PARTNERSHIPS AND THE UK OPPORTUNITY

The panel sessions underscored the extent to which progress in this field will depend on convergence across communities that do not always speak naturally to one another: astronauts, physiologists, clinicians, engineers, investors and translational scientists.

The discussion on biological hurdles in space emphasised that long‐duration missions will require more than incremental problem‐solving; it will require the development and deployment of robust therapeutic interventions and countermeasures. Human missions are already extremely complex. Crew physiological health, cognitive performance and behavioural resilience directly influence mission safety, system reliability and overall exploration capability. Earth‐independent systems will be an order of magnitude more complex.

Radiation exposure, tissue degeneration and operational autonomy remain formidable challenges, but so too does the need to conduct robust, scalable and biologically meaningful experimentation in space. The tone was notably solution‐oriented: the future of space health will depend on interdisciplinary integration rather than siloed expertise (Bailey & van Ombergen, 2026; Bailey et al., 2025; Rutter et al., 2024).

The Babraham Research Campus was therefore a fitting venue, exemplifying how discovery biology, translational science and commercial innovation can be brought into productive alignment. Equally important was the role of coordinated partnerships between academia, industry, investors and national space organisations. The conference reflected the UK Space Agency's commitment to strengthening dialogue between the space and life sciences communities, while the breadth of speakers signalled that the field is now building the partnerships necessary for traction.

As European programmes increasingly incorporate space biology into broader innovation strategies, the UK life sciences sector represents a significant strategic asset. A pertinent recent example outside of Europe that can be drawn upon is the partnership between NASA and Microsoft Federal (Microsoft), alongside Starburst and the Translational Research Institute for Space Health (TRISH), to launch SPACE‐H, a programme designed to vastly accelerate the advancement of strategic healthcare technologies for spaceflight applications. The programme selected 12 global innovations spanning artificial intelligence and computational approaches, synthetic data, engineering, medicine and life sciences. Notably, only two of the 12 selected companies were based outside the United States: LinkGevity, based in the UK, recognised for its anti‐necrotic technology with potential to mitigate accelerated ageing‐related conditions and degradation in astronauts; and Delta Biosciences, based in Lithuania, whose platform supports the preservation of chemical and pharmaceutical integrity in space environments. Both companies participated heavily in this conference. Both companies have subsequently been funded by the UK Space Agency for the collaborative AstroSANITAS programme, ensuring that the anti‐necrotic is space‐stable and positioned to deliver its full operational impact in the near term – thereby underscoring the UK Space Agency's commitment to advancing breakthrough translational innovation and scientific excellence.

This illustrates how established strengths in ageing research, biotechnology, academic medicine and data science can be strategically aligned with space capability to generate both scientific advancement and economic value. The opportunity is substantial; what is now required is not reinvention, but coordinated integration across existing capabilities (Bailey et al., 2025; Mason et al., 2024; Scott et al., 2022) – and for which conferences like this are invaluable.

## 7 A CALL TO ACTION

The discussions in Cambridge made clear that space biology is entering a decisive phase. To realise its potential fully, sustained and strategic support will be required. Policymakers and funders now have an opportunity to ensure that UK researchers and biotech companies remain at the forefront of this rapidly growing field by establishing a dedicated translational space biology programme; fostering public–private partnerships that connect academia, biotech, healthcare systems and space stakeholders; integrating space biology into existing biomedical funding frameworks; supporting integrative physiology and human experimental medicine as core pillars of the field; and investing in platforms that enable autonomous diagnostics, biosensing, biomarker discovery and computational decision support for dual use in astronaut health and terrestrial care.

Such measures would build on existing national strengths, complement current space investments and ensure that UK innovation contributes meaningfully to global space health efforts. Just as importantly, they would help embed the benefits of space‐enabled biomedicine across a broader research and innovation ecosystem rather than concentrating them in only a handful of actors.

## 8 LOOKING AHEAD

As humanity looks towards the Moon, Mars, and beyond, the biological dimension of space exploration is coming into sharp focus. Protecting human health in space will require a deep understanding of how biology responds to extreme environments, and how those responses can be monitored, interpreted and modified (Bailey, 2026; Bailey & van Ombergen, 2026).

What the meeting at the Babraham Research Campus made plain is that space biology is no longer speculative. It is becoming an engine of discovery that links ageing and longevity science, biotechnology and space exploration through shared biological questions. By leveraging the insights gained from space, researchers are not only enabling future exploration, but also advancing new approaches to healthy ageing, disease prevention and resilient healthcare on Earth. In that respect, the most profound legacy of space biology may not be how far it helps humans travel, but how powerfully it helps us understand, protect and extend human function here at home (Bailey, 2026; Camera et al., 2024; Garrett‐Bakelman et al., 2019; Rutter et al., 2024; Scott et al., 2022).

## AUTHOR CONTRIBUTIONS

Damian Miles Bailey wrote the first draft of the manuscript, and other authors contributed equally thereafter. All authors edited the manuscript and approved the final version submitted for publication and agree to be accountable for all aspects of the work in ensuring that questions related to the accuracy or integrity of any part of the work are appropriately investigated and resolved. All persons designated as authors qualify for authorship, and all those who qualify for authorship are listed.

## CONFLICT OF INTEREST

Carina Kern is CEO of LinkGevity. Matthew Cook is Head of Space Exploration, UK Space Agency. Anuj Ashar is General Partner of Asteria Space. Louise Jopling is Chief Scientific & Innovation Officer, Babraham Research Campus Ltd, Cambridge, UK. Immanuel Gartner is Chief Medical Officer, BK Klosterneuburg Dukes, Austria; CEO Gartner.FIT, and Scientific Director, FIBA 3X3, Vienna, Austria. Dominykas Milašius is CEO of Delta Biosciences, Lithuania. Damian Miles Bailey is Editor‐in‐Chief of Experimental Physiology and outgoing Chair of the Life Sciences Working Group and outgoing member of the Human Spaceflight and Exploration Science Advisory Committee to ESA. Damian Miles Bailey is a current member of the ESA‐HRE‐Biology Panel and Space Exploration Advisory Committees to the UK and Swedish National Space Agencies.

## GENERATIVE AI STATEMENT

Generative AI assistance (OpenAI ChatGPT) was used to check and improve the grammar and readability of the manuscript. All scientific content, interpretations, and conclusions were developed and verified by the authors, who take full responsibility for the content of the publication.
